# Roles of end‐binding 1 protein and gamma‐tubulin small complex in cytokinesis and flagella formation of *Giardia lamblia*

**DOI:** 10.1002/mbo3.748

**Published:** 2018-10-14

**Authors:** Juri Kim, Soon‐Jung Park

**Affiliations:** ^1^ Department of Environmental Medical Biology and Institute of Tropical Medicine, Brain Korea 21 PLUS Project for Medical Science Yonsei University College of Medicine Seoul South Korea

**Keywords:** EB1, flagella, *Giardia lamblia*, median body, γ‐tubulin small complex

## Abstract

*Giardia lamblia* is a unicellular organism with two nuclei, a median body, eight flagella, and an adhesive disk. γ‐Tubulin is a microtubule (MT)‐nucleating protein that functions in the γ‐tubulin small complex (γ‐TuSC) in budding yeast. In this study, *G. lamblia* γ‐tubulin (Glγ‐tubulin) was found to bind to another MT‐binding protein, namely *G. lamblia* end‐binding protein 1 (GlEB1), via both in vivo and in vitro assays. Hemagglutinin (HA)‐tagged Glγ‐tubulin localized to the basal bodies, axonemes, and median bodies of *G. lamblia* trophozoites. The knockdown of Glγ‐tubulin expression using an anti‐Glγ‐tubulin morpholino resulted in a decreased growth rate and an increased failed cytokinesis cells of *Giardia*. The formation of median bodies was affected, and the central pair of MTs in flagella was frequently missing in the *Giardia* treated with an anti‐Glγ‐tubulin morpholino. *G. lamblia* γ‐tubulin complex protein 2 (GlGCP2) and GlGCP3, which are putative components of γ‐TuSC, were co‐immunoprecipitated with HA‐tagged Glγ‐tubulin in *Giardia* extracts. The knockdown of GlGCP2 and GlGCP3 expression also resulted in decreased formation of both the median body and flagella MTs. Knockdown of Glγ‐tubulin, GlGCP2, and GlGCP3 expression affected localization of GlEB1 in *G. lamblia*. In addition, decreased level of GlEB1 caused reduced formation of median body and the central pair of flagella MTs. These results indicated that Glγ‐tubulin plays a role in MT nucleation for median body formation and flagella biogenesis as a component of Glγ‐TuSC in *Giardia* and GlEB1 may be involved in this process.

## 1 INTRODUCTION

Microtubules (MTs) are essential components of the cytoskeletons in eukaryotic organisms and play roles in organelle positioning and intracellular transport (Nogales, 2001). Plus‐end tracking proteins (+TIPs) associating with the growing ends of polymerized MTs mediate the polymerization and depolymerization of MTs (Akhmanova & Steinmetz, 2008). End‐binding 1 (EB1) protein is one of the main components of +TIPs, as shown for the yeast EB1 homologs, Mal3p (Busch, Hayles, Nurse, & Brunner, 2004), and Bim1p (Zimniak, Stengl, Mechtler, & Westermann, 2009), as well as the mammalian EB1 (Komarova et al., 2009).

*Giardia lamblia* is a unicellular organism that has bilateral symmetry from the side view and polarity in the anterior/posterior and dorsal/ventral directions. *G. lamblia* cells have unique cytoskeletal structures, such as four pairs of flagella, a median body, and an adhesive disk (Elmendorf, Dawson, & McCaffery, 2003). The exact positioning of these organelles should be regulated via the proper function of MTs when this organism divides (Desai & Mitchison, 1997). *G. lamblia* EB1 (GlEB1) was found at the flagella tips, median bodies, nuclear membranes, and mitotic spindles (Dawson et al., 2007; Kim, Nagami, Lee, & Park, 2014). GlEB1 was also found to complement a *BIM1* mutant of *Saccharomyces cerevisiae*, that is, to induce the proper positioning of the nucleus (Kim et al., 2008). In vitro studies have demonstrated that GlEB1 can be phosphorylated by *G. lamblia* aurora kinase (GlAK) (Kim, Lee, Lee, & Park, 2017). The ectopic expression of a mutant GlEB1 in which Ser148 was changed to Ala resulted in an increased number of *Giardia* cells with division defects. The treatment of *G. lamblia* with an aurora kinase inhibitor triggered cytokinesis defects, and the ectopic expression of a phospho‐mimetic mutant GlEB1 in which Ser148 was changed to Asp rescued the defects in *Giardia* cell division caused by that inhibitor, even though it has not yet been determined whether GlEB1 is a direct substrate of GlAK.

In *S. cerevisiae*, a mutant lacking the C‐terminal four residues of γ‐tubulin was defective in the proper recruitment of the Kar9p‐Bim1p complex (Cuschieri, Miller, & Vogel, 2006). In addition, the overexpression of the EB1 ortholog Bim1p, but not Kar9p, rescued the mutant with defective γ‐tubulin. In this study, we examined whether GlEB1 and *G. lamblia* γ‐tubulin (Glγ‐tubulin) had any functional relationship in *G. lamblia* by measuring their physical association.

γ‐Tubulin, which presents a specialized member of the tubulin family, is a MT‐nucleating protein localized at MT‐organizing centers (MTOCs) in eukaryotes (Gull, 1999). It exists as a complex called γ‐tubulin small complex (γ‐TuSC) with γ‐tubulin complex protein (GCP) 2 and GCP3 in a molar ratio of 2:1:1 (Knop, Pereira, Geissler, Grein, & Schiebel, 1997). In organisms of higher complexity, γ‐TuSC becomes a component of the γ‐tubulin ring complex (γ‐TuRC) with additional subunits named GCP4–6 (Kollman, Merdes, Mourey, & Agard, 2011) and non‐GCP family proteins (Hutchins et al., 2010; Teixidó‐Travesa et al., 2010). Little information is available on the γ‐tubulin of *G. lamblia* (Nohynková, Draberb, Reischigc, & Kulda, 2000). While Glγ‐tubulin was mainly found in the basal bodies/axonemes of flagella in *G. lamblia* cells under all the stages, this protein is transiently localized in the centers of mitotic spindles only in the dividing cells. In this study, we examined the roles of γ‐tubulin and GCPs in MT modulation in *G. lamblia*.

## 2 MATERIALS AND METHODS

### 2.1 *Giardia* strain and cultivation

*Giardia lamblia* WB strain (ATCC 30957; American Type Culture Collection, Manassas, VA) were cultured at 37°C as described in the previous paper (Kim et al., 2014).

### 2.2 In vitro co‐immunoprecipitation assays

The interaction between GlEB1 and Glγ‐tubulin was monitored by using the BD Matchmaker Co‐IP Kit (Clontech, Mountain View, CA). The pGBKEB1 produced Myc‐tagged GlEB1 protein (Kim et al., 2017). A 1,476‐bp DNA fragment encoding Glγ‐tubulin was cloned into pGADT7 (Clontech) to produce pGADγ‐tubulin, in which Glγ‐tubulin was expressed in an hemagglutinin (HA)‐tagged form. [^35^S]methionine‐labeled Myc‐tagged GlEB1 and HA‐tagged Glγ‐tubulin were synthesized in vitro using the TNT^®^ Coupled Reticulocyte Lysate Systems (Promega, Madison, WI). These two proteins were mixed in two separate tubes; monoclonal antibodies for the Myc epitope were added into one tube, while polyclonal antibodies specific for the HA epitope were added to the other tube. These antibodies/labeled protein complexes were precipitated with protein A beads. The eluted proteins were separated by 12% sodium dodecyl sulfate polyacrylamide gel electrophoresis (SDS‐PAGE) and observed using autoradiography.

### 2.3 Glutathione *S*‐transferase (GST) pull‐down assays

The full length of recombinant GST‐tagged GlEB1 (GST‐GlEB1) protein was expressed in *Escherichia coli* BL21 (DE3) carrying pGEXEB1^1–238^ (Kim et al., 2017) with 0.5 mM isopropyl β‐ᴅ‐1‐thiogalactopyranoside (IPTG) and purified using glutathione Sepharose^®^ 4B affinity chromatography (GE Healthcare, Uppsala, Sweden). GST protein was also purified as described above and used as a control. Glutathione Sepharose^®^ 4B resin coupled with 5 µg of either purified GST or the GST‐GlEB1 protein was incubated with an *E. coli* lysate expressing His‐tagged Glγ‐tubulin in a binding buffer (20 mM Tris–HCl, 500 mM NaCl, 0.1% Triton X‐100, pH 7.5). After an overnight incubation at 4°C, the resins were washed three times with washing buffer (10 mM Tris–HCl, 150 mM NaCl, 0.1% Triton X‐100, pH 7.5) and eluted for western blot analysis.

### 2.4 Yeast two‐hybrid assays

The *S. cerevisiae* AH109 strain carrying dual reporter systems, namely *GAL1* promoter—*HIS3* and *MEL1* promoter—*lacZ* (Vojtek, Hollenberg, & Cooper, 1993), was grown at 30°C. For plates to induce expression of *GAL4* gene, 2% galactose and 1% raffinose were added instead of 2% glucose.

### 2.5 Construction of HA epitope‐tagged Glγ‐tubulin and HA‐tagged GlGCP3′

An 1,676‐bp DNA fragment of the *gl*γ‐*tubulin* gene, which is composed of the promoter region near the N‐terminus and an HA epitope at the C‐terminus of the ORF, was amplified from *Giardia* genomic DNA by polymerase chain reaction (PCR) using two primers, namely γ‐tubulin‐NcoI‐F and γ‐tubulin‐HAX3‐R (Table 1). The NcoI and NotI sites were used for cloning into the plasmid pGFP.pac (Singer, Yee, & Nash, 1998), resulting in the plasmid pGlγ‐tubulinHAX3.pac. The construct was confirmed by DNA sequencing by a sequencing service company (Macrogen, Seoul, Korea).

**Table 1:** Primers and morpholino used in this study

Name	Nucleotide sequence (5'−3')[Link] ^,^ [Link]	
Co‐immunoprecipitation assay		
COIPGAD‐114218‐F		CCGGAATTCATGTGCGTTTATATTGAA
COIPGAD‐114218‐R		CCGCTCGAGCCATCCCGATATACTCAAG
Recombinant Glcentrin protein		
rcentrin‐F‐NcoI		CATGCCATGGGCATGAATAGAGCGGCCATAG
rcentrin‐His‐R‐NotI		ATAGTTTAGCGGCCGCAGAGAAAGCACTTGTGGAC
Recombinant Glγ‐tubulin protein		
rγ‐tubulin‐F‐ERI		GATCGAATTCGATGTGCGTTTATATTGAAAATT
rγ‐tubulin‐R‐XhoI		CCGCTCGAGCTTGGGGACCTTACTCCTGT
Transgenic *G. lamblia* expressing HA‐tagged Glγ‐tubulin		
γ‐tubulin‐NcoI‐F		CATCCCATGGTTAGGATGAGGCGGATGGTGTAG
γ‐tubulin‐HAX3‐R		GTTACGCGGCCGCTTA*AGCGTAATCTGGAACATCGTATGGGTAAGCGTAATCTGGAACATCGTATGGGTAAGCGTAATCTGGAACATCGTATGGGTA*CATCCCGATATACTCAAGGTAGT
Mopholino sequences		
Control		CCTCTTACCTCAGTTACAATTTATA
Anti‐Glγ‐tubulin		CAATATAAACGCACATTGCGAAGAG
Anti‐GlGCP2		GTCCACCCTGAAACACATACGCATG
Anti‐GlGCP3		GTAAAGCGAGGATTGTCTCTAGCAT
Anti‐GlEB1		TTCCGGGTGCTTTTACCGGCGGCAT
Transgenic *G. lamblia* expressing HA‐tagged GlGCP3		
GCP3‐SacI‐F		GGCGAGCTCTGCGTTTACCATTCCAAGGTCT
GCP3‐HAX3‐part‐R		GTTACGCGGCCGCTTA*AGCGTAATCTGGAACATCGTATGGGTAAGCGTAATCTGGAACATCGTATGGGTAAGCGTAATCTGGAACATCGTATGGGTA*ACTGATGTAATTAAGCAAAGCAAG

Restriction enzyme sites are underlined.

Mutated bases are indicated as italic letters.

A 2,220‐bp DNA containing 100‐bp of promoter region of *glgcp3* gene and partial GlGCP3 ORF (amino acid #1–#740) was amplified from *G. lamblia* genomic DNA by PCR using primer GCP3‐SacI‐F and GCP3‐HAX3‐part‐R (Table 1). The resulting DNA was cloned into pRAN.neo (Sun, Chou, & Tai, 1998), producing pGlGCP3HAX3part.neo.

Thirty micrograms of pGlγ‐tubulinHAX3.pac or pGlGCP3HAX3part.neo was transformed into 1 × 10^7^ trophozoites by electroporation under the following conditions: 350 V, 1,000 µF, and 700Ω (Bio‐Rad, Hercules, CA). After electroporation, cells harboring the plasmid were selected by 50 µg/ml of puromycin (A. G. Scientific, San Diego, CA) or 600 µg/ml of G418 (A. G. Scientific). As a control, *Giardia* trophozoites carrying pΔ.pac or pRAN.neo were also made as described above. The control plasmid, pΔ.pac, was created by deleting the *gfp* genes in pGFP.pac.

### 2.6 Western blot analysis

Protein lysates were prepared from *G. lamblia* carrying pGlγ‐tubulinHAX3.pac, pGlGCP3HAX3part.neo, pΔ.pac, or pRAN.neo in a phosphate‐buffered saline (PBS: 137 mM NaCl, 2.7 mM KCl, 10.1 mM Na_2_HPO_4_, and 2 mM KH_2_PO_4_, pH 7.4). The separated extracts transferred onto a polyvinylidene fluoride membrane (Millipore, Bedford, MA), and the membrane was then reacted overnight with monoclonal mouse anti‐HA (1:1,000; Sigma‐Aldrich, St. Louis, MO) in a blocking solution (50 mM Tris‐HCl, 5% skim milk, and 0.05% Tween 20) at 4°C. Next, the blot was incubated with horseradish peroxidase‐conjugated secondary antibodies and the immunoreactive protein was detected using an enhanced chemiluminescence system (Thermo Scientific, Rockford, IL). The blots were then incubated in a stripping buffer (Thermo Scientific) for 30 min and reacted with polyclonal rat antibodies specific to the protein disulfide isomerase (PDI) 1 of *G. lamblia* (1:10,000) (Kim et al., 2017).

### 2.7 Co‐immunoprecipitation of GlEB1 or GlGCP2 or GlGCP3 and Glγ‐tubulin with anti‐HA antibodies from *Giardia* carrying pGlγ‐tubulinHAX3.pac

*Giardia* cells expressing HA‐tagged Glγ‐tubulins were harvested and resuspended in ice‐cold cross‐linking buffer (50 mM Tris–HCl, pH 7.4, and 150 mM NaCl) supplemented with a protease inhibitor cocktail (Thermo Scientific). After incubation for 1 hr on ice, these cells were lysed by sonication with 2‐s pulses at 20% amplitude (Pronextech, Seoul, Korea). The supernatants were obtained by centrifugation at 16,000 x g for 20 min and were then precleared with protein A/G beads (Pierce, Waltham, MA) for 1 hr at 4°C. One milligram of each lysate was reacted with anti‐HA agarose beads (Sigma‐Aldrich) at 4°C overnight. After two washes with cross‐linking wash buffer (50 mM Tris‐HCl, pH 7.4, 150 mM NaCl, and 1% Triton X‐100), the beads were resuspended with SDS‐loading buffer and boiled for 5 min. Twenty micrograms of the eluted fraction was analyzed by western blotting using anti‐HA (Sigma‐Aldrich) to monitor the presence of HA‐tagged Glγ‐tubulin. The same sample was treated with antibodies against Glγ‐tubulin, GlEB1 (Kim et al., 2017), GlGCP2, or GlGCP3 antibodies. As a control, *Giardia* caring HA‐tagged Glγ‐tubulin extracts were incubated with anti‐mouse IgG conjugated to Sepharose^®^ beads (Cell Signaling, Danvers, MA) instead of anti‐HA agarose beads.

### 2.8 Formation of antibodies specific to *G. lamblia* centrin (Glcentrin), Glγ‐tubulin, GlGCP2, and GlGCP3 proteins

For making of recombinant Glcentrin (rGlcentrin), a 531‐bp *glcentrin* DNA fragment (GiardiaDB ORF No: GL50803_104685) made with two primers, rcentrin‐F‐NcoI and rcentrin‐His‐R‐NotI (Table 1), was cloned into pET28b to produce pETcentrin (Table 2).

**Table 2:** Strains and plasmids used in this study

Organism/Plasmid	Description[Link]	Source/Reference
*Giardia lamblia*		
ATCC 30957	Clinical isolate	ATCC
*Escherichia coli*		
DH5α	*supE44, ΔlacU169 (Φ80 lacZ ΔM15), hsdR17, recA1, endA1, gyrA96, thi‐1, relA1*	Invitrogen
BL21 (DE3)	*F′, ompT, hsdS_B_(r_B_^‐^m_B_^‐^) gal, dcm (DE3)*	Invitrogen
*Saccharomyces cerevisiae*		
AH109	*MATα, ura3, his3, trp1, lexAop(X6)‐LEU2*, p8op‐*lacZ*	Clontech
Plasmids		
pGBKT7	Gal4p_(1__–__147)_ DNA‐BD, *TRP1,* Kan^R^, c‐Myc Epitope	Clontech
pGBK‐p53	pGBKT7, murine p53_(72–390)_	Clontech
pGBK‐Lam	pGBKT7, human lamin C_(66–330)_	Clontech
pGBKEB1	pGBKT7, 717‐bp encoding *gleb1*	Kim et al. (2017)
pGADT7	Gal4p_(768__–__881)_‐AD, *LUE2,*Amp^R^, HA epitope	Clontech
pGAD‐T	pGADT7, SV40 large T‐antigen_(87–708)_	Clonetech
pGADγ‐tubulin	pGADT7, 1,476‐bp encoding *glγ‐tubulin*	This study
pGEX4T‐1	Expression vector, Amp^R^, GST	GE Healthcare
pGEXEB1^1–238^	pGEX4T‐1, 717‐bp encoding *gleb1*	Kim et al. (2017)
pET28b	Expression vector, Kan^R^	Novagen
pETcentrin	pET28b, 531‐bp encoding *glcentrin*	This study
pET32b	Expression vector, Amp^R^	Novagen
pETγ‐tubulin	pET32b, 1,476‐bp encoding *glγ‐tubulin*	This study
pGFP.pac	Shuttle vector, Amp^R^, *pac* gene	Singer et al. (1998)
pGlγ‐tubulinHAX3.pac	pGFP.pac, 1,676‐bp encoding *glγ‐tubulin* with its own promoter	This study
pRAN.neo	Shuttle vector, Amp^R^, *neo* gene	Sun et al. (1998)
pGlGCP3HAX3part.neo	pRAN.neo, 2,320‐bp encoding *glgcp3* with its own promoter	This study

Amp: ampicillin; Kan: kanamycin; ^R^: resistant; DNA‐BD: DNA‐binding domain; AD‐activation domain; HA: hemagglutinin.

The primers used to make recombinant Glγ‐tubulin (rGlγ‐tubulin) were made based on GiardiaDB ORF No: GL50803_114218. A 1,476‐bp DNA fragment was amplified by PCR using the two primers rγ‐tubulin‐F‐ERI and rγ‐tubulin‐R‐XhoI (Table 1). The amplified fragment was then cloned into pET32b (Novagen, Darmstadt, Germany) to obtain the plasmid pETγ‐tubulin (Table 2).

Histidine‐tagged rGlcentrin or Glγ‐tubulin was expressed in *E. coli* BL21 (*DE3*) by adding of 1 mM IPTG and then used to immunize Sprague–Dawley rats (2‐week‐old, female) to make polyclonal antibodies.

To generate antibodies specific to GlGCP2 (GiardiaDB ORF No; GL50803_17429) and GlGCP3 (GiardiaDB ORF No; GL50803_12057), the prediction of the antigenic region was performed by Young In Frontier (Seoul, Korea). Two antigen peptides, namely GlGCP2 (amino acids 909–927: NEESREGKSGPRGVKGSER) and GlGCP3 (amino acids 121–139: KTNKLHGKSKHKSKKSIRSC), were designed. Two peptides (5 mg each) were synthesized, conjugated to keyhole limpet hemocyanin, and then used for immunizing mice or rabbit (Young In Frontier).

These polyclonal antibodies were purified using protein A or protein G resin to obtain specific IgG. The specificity of the purified antibodies was confirmed by a western blot analysis of *Giardia* extracts.

### 2.9 Immunofluorescence assay (IFA)

To monitor the localization of Glγ‐tubulin and MTs in *G. lamblia* expressing HA‐tagged Glγ‐tubulin, *G. lamblia* on glass slides were fixed with chilled 100% methanol for 10 min at −20°C and followed by permeabilization with PBS/0.5% Triton X‐100 for 10 min at room temperature. The cells were incubated in blocking buffer (PBS, 5% goat serum, and 3% bovine serum albumin) for 1 hr and then reacted with rat anti‐HA antibodies (rat monoclonal antibodies, 1:50; clone 3F10, Roche, Indianapolis, IN) and anti‐α‐tubulin antibodies (mouse monoclonal anti‐acetylated α‐tubulin antibodies, 1:800; clone 6‐11B‐1, Sigma‐Aldrich) overnight. After washing three times for 5 min with PBS, the cells were reacted with AlexaFluor 488‐conjugated anti‐rat IgG and AlexaFluor 564‐conjugated anti‐mouse IgG (1:100; Molecular Probes, Grand Island, NY) at 37°C for 1 hr. The cells were mounted with VECTASHIELD anti‐fade mounting medium containing 4′,6‐diamidino‐2‐phenylindole (DAPI; Vector Laboratories, Burlingame, CA). They were observed with an LSM 710 laser scanning confocal microscope (Carl Zeiss, Oberkochen, Germany). Images were acquired with serial sections at 0.3 μm intervals and created maximum‐intensity projection using Zeiss ZEN 2011 image browser software (Carl Zeiss).

To examine co‐localization of Glγ‐tubulin with Glcentrin, a marker of MTOC (Lauwaet et al., 2011), *Giardia* cells carrying pGlγ‐tubulinHAX3.pac were stained with anti‐HA (1:50) and anti‐Glcentrin antibodies (1:100).

*G. lamblia* expressing HA‐tagged Glγ‐tubulin or *G. lamblia* expressing HA‐tagged GlGCP3 was used to examine co‐localization of Glγ‐tubulin and GlGCP2 or co‐localization of GlGCP2 and GlGCP3, respectively.

In addition, *Giardia* expressing HA‐tagged GlEB1 was double‐stained with anti‐HA (1:50) and anti‐α‐tubulin antibodies (1:800) as described above in order to examine localization of GlEB1 and MTs.

### 2.10 Morpholino knockdown of Glγ‐tubulin, GlGCP2, GlGCP3, or GlEB1 expression

Decreased Glγ‐tubulin expression was observed by a knockdown experiment using morpholino as described (Carpenter & Cande, 2009). The cells were treated with 25‐mer morpholino for Glγ‐tubulin, which included 16 nucleotides of the Glγ‐tubulin ORF and nine nucleotides upstream of the start codon (Table 1; Gene Tools, Philomath, OR). The non‐specific oligomers provided by the company were used as a controls morpholino (Table 1). The lyophilized morpholino was added to 5 × 10^6^ cells in 0.3 ml medium at a final concentration of 100 or 200 μM. As another negative control, an equal volume of sterile water was added to the cells. After electroporation, the cells were grown for 24 or 48 hr and then analyzed for the expression of Glγ‐tubulin by western blot as described above. The specific morpholino for GlGCP2 and GlGCP3 was also designed by Gene Tools, and their sequences are listed in Table 1.

### 2.11 Measuring the growth and cell division of *G. lamblia* trophozoites

After 24 hr post‐treatment with morpholino, the number of parasites per milliliter was determined using a hemocytometer. With *G. lamblia* trophozoites treated with water, a control morpholino, or the anti‐Glγ‐tubulin morpholino, the proportions of cells with two or four nuclei were determined to monitor cytokinesis as previously described (Hofstetrova et al., 2010). The cells were attached onto glass slides, fixed with methanol, air‐dried, and then mounted in VECTASHIELD anti‐fade mounting medium containing DAPI (Vector Laboratories). The cells with two or four nuclei in each condition were counted among a total of over 300 cells.

Effect of knockdown of Glγ‐tubulin, GlGCP2, and GlGCP3 on cytokinesis was monitored as described (Hardin et al., 2017). Various *G. lamblia* trophozoites (controls, and the cells treated with anti‐Glγ‐tubulin, anti‐GlGCP2, and anti‐GlGCP3 morpholino) were grouped by the following phenotypes: disorganized for cytokinesis, defective in furrow formation, disable in cytokinesis, and failed abscission.

### 2.12 Determination of phenotypes related to the median body and flagella of *G. lamblia*

For *Giardia* cells treated with water, control, or anti‐Glγ‐tubulin morpholino, the percentage of cells having a median body was determined by Giemsa staining. The cells were attached onto glass slides, air‐dried, and then fixed with 100% methanol for 10 min. They were then stained with 10% Giemsa solution for 40 min and washed with distilled water. After mounting with dibutyl phthalate xylene mountant (Sigma‐Aldrich), the slides were observed with an Axiovert 200 microscope (Carl Zeiss).

To measure the volume of the median bodies, the morpholino‐treated cells were stained with 6‐11B‐1, which is the monoclonal antibodies against α‐tubulin (1:600; Sigma‐Aldrich), followed by a reaction with AlexaFlour 488‐conjugated anti‐mouse IgG (1:200; Molecular Probes). The IFA procedure was the same as described above. Samples were observed with an LSM710 laser scanning confocal microscope (Carl Zeiss), and serial sections were acquired at 0.3 μm intervals. For the measurement of median body volume, image analysis was performed using Imaris (Bitplane, South Windsor, CO).

*G. lamblia* cells stained with 6–11B‐1 were also used to observe the effect of morpholino on flagella formation. Specifically, the length of the caudal flagella was measured using Zen 2012 (the blue edition, Carl Zeiss).

### 2.13 Transmission electron microscopy (TEM)

For TEM, morpholino‐treated cells were fixed with 2% glutaraldehyde‐2% paraformaldehyde in 0.1 M phosphate buffer (pH 7.4). They were post‐fixed with 1% OsO_4_ in 0.1 M phosphate buffer (pH 7.4) for 2 hr, dehydrated in an ascending graduated series (50%–100%) of ethanol, and infiltrated with propylene oxide. Specimens were then embedded using a Poly/Bed 812 kit (Polysciences, Warrington, PA). After embedding, the specimens were polymerized at 65°C in the electron microscope oven (TD‐700, DOSAKA EM, Kyoto, Japan) for 24 hr. Thin sections (70 nm thickness) were double‐stained with 6% uranyl acetate and lead citrate (Fisher Scientific, Rockford, IL) for contrast staining. The sections were cut using an EM UC‐7 microtome (Leica Microsystems, Seoul, Korea) with a diamond knife and then transferred onto copper grids. All the thin sections were observed by TEM (JEM‐1011, JEOL, Seoul, Korea) at the acceleration voltage of 80 kV.

### 2.14 Statistical analysis

Data are presented as the mean ± standard deviation from three independent experiments. To determine the statistical significance of these results, data were performed using Student's *t* tests by statistical analyses for pair‐wise comparisons. Differences with *p*‐values of <0.05 were considered significant. In the figures and tables, two asterisks indicate *p*‐values of <0.01, while a single asterisk indicates *p*‐values between 0.01 and 0.05.

## 3 RESULTS

### 3.1 Interaction of Glγ‐tubulin with GlEB1

**Figure 1:**
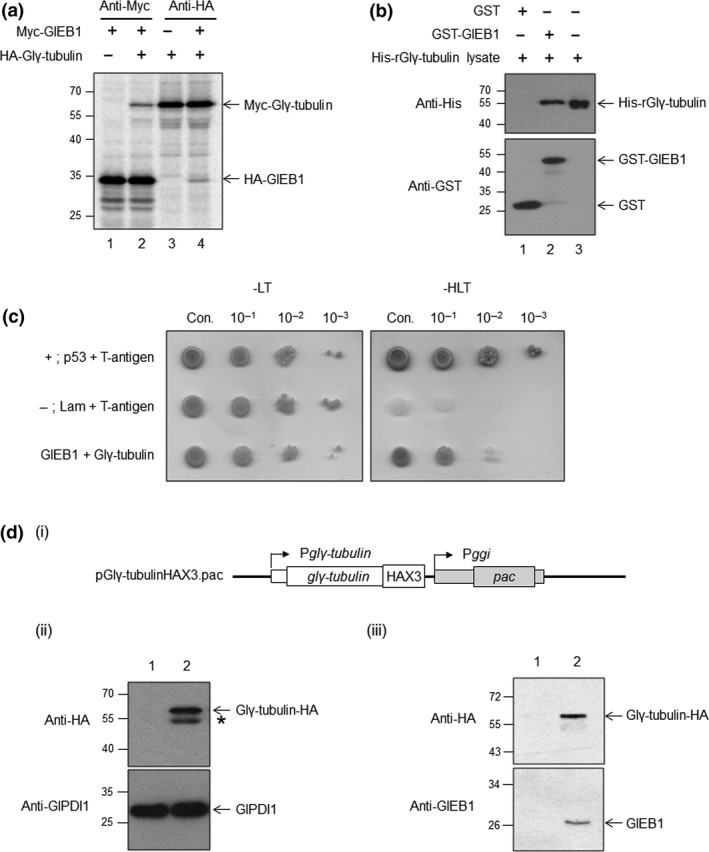
Interaction of Glγ‐tubulin with GlEB1. (a) Co‐immunoprecipitation of Glγ‐tubulin with GlEB1. An in vitro‐synthesized a Myc‐tagged GlEB1 was precipitated with anti‐Myc antibodies (lane 1), while Glγ‐tubulin with a HA epitope was sedimented with anti‐HA antibodies (lane 3). Myc‐tagged GlEB1 and HA‐tagged Glγ‐tubulin mixture were precipitated either anti‐Myc or anti‐HA antibodies (lanes 2 and 4, respectively). (b) Purified GST‐tagged GlEB1 proteins were incubated with *E. coli* lysates expressing histidine‐tagged Glγ‐tubulin, and these proteins were precipitated with glutathione Sepharose resin (lane 2). As a control, GST was prepared and incubated with *E. coli* lysates expressing histidine‐tagged Glγ‐tubulin (lane 1) and lysates expressing the histidine‐tagged Glγ‐tubulin was loaded (lane 3). The precipitated proteins were analyzed by western blotting using anti‐GST or anti‐histidine antibodies. The immunoreactive GST, Glγ‐tubulin, and GST‐GlEB1 proteins are indicated by arrows. (c) The yeast two‐hybrid assay. A serial dilution of yeast cells was spotted on selective indicator plates. Yeast cells bearing pGBK‐p53 and pGAD‐T were used as a positive control for interaction, while yeast carrying pGBK‐Lam and pGAD‐T were used as a negative control. L: leucine; T: tryptophan; H: histidine (d) Co‐immunoprecipitation of Glγ‐tubulin with GlEB1 from HA‐tagged Glγ‐tubulin expressing *G. lamblia* lysates. (i) A schematic diagram of the plasmid pGlγ‐tubulinHAX3.pac. Glγ‐tubulin is expressed from its own promoter, P*glγ‐tubulin*, as a HA‐tagged form (HAX3). Transfected cells are selected by puromycin resistance conferred by the *pac* gene expressed by the P*ggi* promoter, a promoter of the γ‐giardin protein gene. (ii) The expression of HA‐tagged Glγ‐tubulin was confirmed by western blot analysis. Extracts were prepared from *G. lamblia* containing pΔ.pac (lane 1) or pGlγ‐tubulinHAX3.pac (lane 2). The membrane was reacted with monoclonal mouse anti‐HA (1:1,000). After deprobing in the stripping buffer, the membrane was incubated with polyclonal rat antibodies specific to PDI1 of *G. lamblia* (1:10,000) as loading control. (iii) Co‐immunoprecipitation of Glγ‐tubulin with GlEB1 from *Giardia* carrying pGlγ‐tubulinHAX3.pac. Cell extracts containing HA‐tagged Glγ‐tubulin proteins were pre‐cleared with protein A/G beads. As a control, *Giardia* extracts were incubated with Sepharose bead‐conjugated anti‐mouse IgG (lane 1). One milligram of lysates was reacted with anti‐HA agarose beads at 4°C overnight (lane 2). Twenty micrograms of the eluted fraction was analyzed by western blot using anti‐HA or anti‐GlEB1 antibodies

In *S. cerevisiae*, γ‐tubulin was found to be involved in the function of Bim1p, which is orthologous to EB1 (Cuschieri et al., 2006). A homology search in the *Giardia* database indicated an ORF, GL50803_114218, as a putative Glγ‐tubulin. Subsequently, two in vitro assays were performed to examine whether this Glγ‐tubulin interacted with GlEB1. Using an in vitro transcription/translation system, GlEB1 and Glγ‐tubulin were produced in the Myc‐tagged and HA‐tagged forms, respectively. Each protein was precipitated with corresponding antibodies (Figure 1a, lanes 1 and 3). When these two proteins were mixed, and incubated with one of the antibodies, both Glγ‐tubulin and GlEB1 were present in the precipitates with Myc or HA antibodies (Figure 1a, lanes 2 and 4, respectively).

In the subsequent experiment, GST‐tagged GlEB1 protein, GST‐GlEB1, was examined to determine whether it could interact with histidine‐tagged Glγ‐tubulin (Figure 1b). Glγ‐tubulin was found to bind to the glutathione resin coupled with GST‐GlEB1, whereas incubation with GST alone did not result in the precipitation of rGlγ‐tubulin (Figure 1b, lanes 2 and 3, respectively).

Using an yeast system, we confirmed the interaction between Glγ‐tubulin and GlEB1 in vivo. The pGADγ‐tubulin was constructed to produce Glγ‐tubulin with the activation domain of yeast Gal4p. The AH109 strain transformed with pGBKEB1 and pGADγ‐tubulin demonstrated an interaction‐positive phenotype, that is, growth on the indicator plates lacking histidine, leucine, and tryptophan (Figure 1c). Similarly, the AH109 clones with pGBK‐p53 and pGAD‐T show an interaction‐positive phenotype on the indicator plates because of the interaction between the T‐antigen and p53. However, the AH109 clones with pGBK‐Lam and pGAD‐T, which represented a negative control lacking any interaction, showed no growth on the indicator plates.

In this study, pGlγ‐tubulinHAX3.pac was made (Figure 1d(i)) and then used to construct transgenic trophozoites expressing the HA‐tagged Glγ‐tubulin. The expression of the HA‐tagged Glγ‐tubulin as an immunoreactive band of 59 kDa was confirmed by a western blot analysis of the resulting *G. lamblia* extracts (Figure 1d(ii)). In contrast, extracts of *G. lamblia* carrying the vector control, pΔ.pac, did not produce any immunoreactive bands in the same western blot analysis. Western blot of the same membrane with anti‐GlPDI1 antibodies (Kim et al., 2017) served as a loading control for the total amount of protein in the extracts used for this assay.

We performed an additional assay to examine whether GlEB1 could be precipitated with anti‐HA antibodies from this transgenic *G. lamblia* expressing the HA‐tagged Glγ‐tubulin (Figure 1d(iii)). Extracts of the HA‐tagged Glγ‐tubulin expressing cells were incubated with a resin coupled with anti‐HA antibodies. As a control, the same extracts were bound to a resin coupled with mouse IgG. The eluted fractions were analyzed by western blots using anti‐HA or anti‐GlEB1 antibodies. Only the eluted proteins obtained from the resin coupled to anti‐HA (lane 2) demonstrated the presence of the HA‐tagged Glγ‐tubulin and GlEB1. The eluent from the mouse IgG resin (lane 1) did not show any immunoreactive band in the western blot analysis.

### 3.2 Determination of localization of Glγ‐tubulin during cell cycle of the *Giardia*

**Figure 2:**
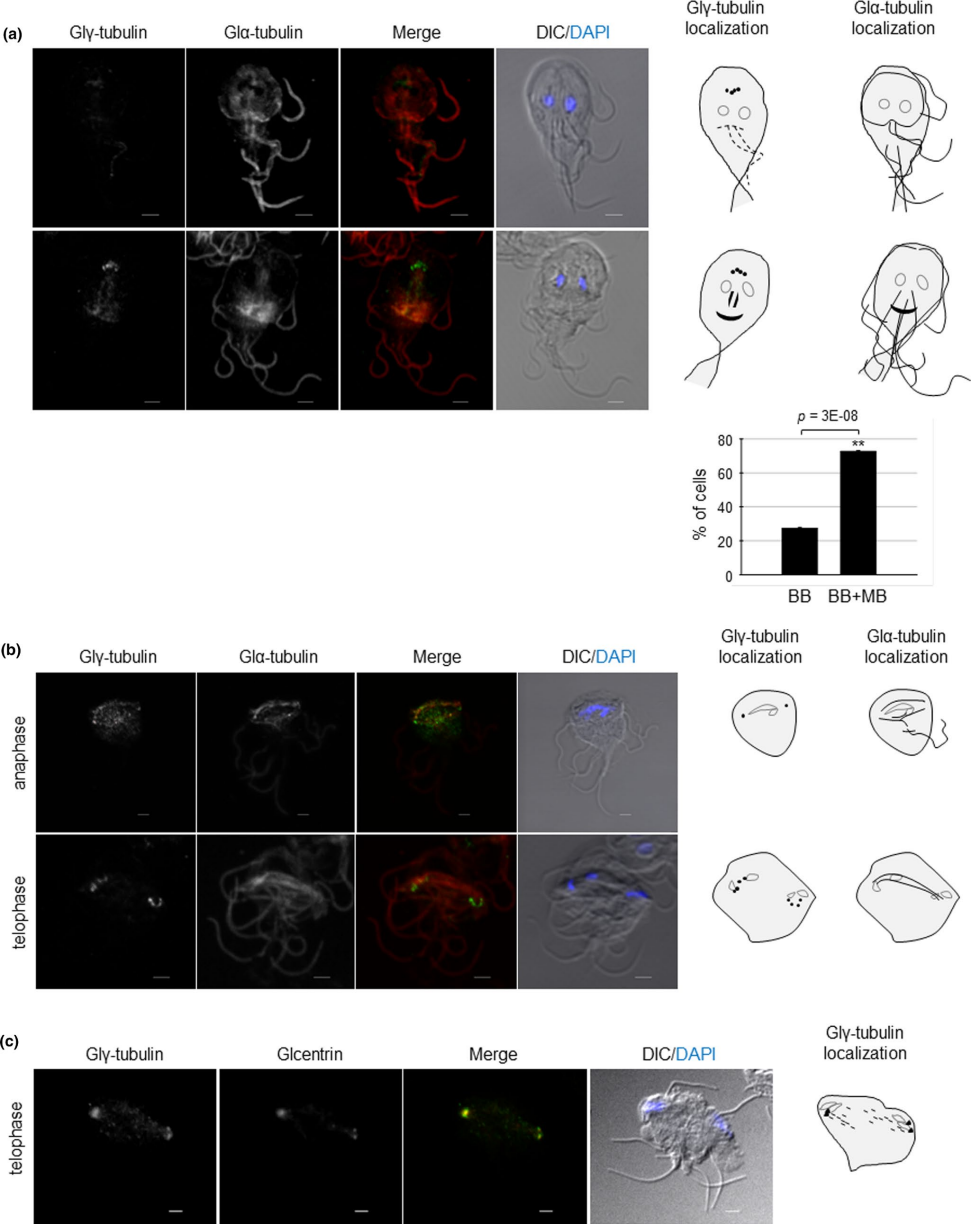
Localization of Glγ‐tubulin and Glα‐tubulin in *G. lamblia* expressing HA‐tagged Glγ‐tubulin. *G. lamblia* expressing HA‐tagged Glγ‐tubulin attached to glass slides were reacted overnight with rat anti‐HA (1:100) and anti‐α‐tubulin antibodies (1:800). The cells were then incubated with AlexaFluor 488‐conjugated anti‐mouse IgG (1:100) and AlexaFluor 564‐conjugated anti‐mouse IgG (1:100). The cells were mounted with DAPI containing anti‐fade mounting medium and observed using an LSM710 laser scanning confocal microscope (Carl Zeiss). A DIC image was acquired to show the cell morphology. (a) Localization of Glγ‐tubulin and Glα‐tubulin in *G. lamblia* trophozoites at interphase. Percentages of *Giardia* cells without or with median bodies are indicated in the bar graph. BB: basal body; MB: median bodies. (b) Localization of Glγ‐tubulin and Glα‐tubulin in *G. lamblia* trophozoites undergoing cell division. (c) Localization of Glγ‐tubulin and Glcentrin in *Giardia* at telophase. The nuclei are indicated by gray lines, and Glγ‐tubulin and MTs are represented as black spots or lines in the cartoons of *Giardia* trophozoites. All the images are maximum‐intensity projections. Scale bar: 2 μm

*Giardia* expressing HA‐tagged Glγ‐tubulin were double‐stained for Glγ‐tubulin and MTs using anti‐HA and anti‐α‐tubulin antibodies, respectively. In most of *Giardia* trophozoites at interphase, Glγ‐tubulin was mainly found in the basal bodies. We observed weak fluorescence signals at a portion of the margins of adhesive disks and the ventral flagella. Some portion (63%) of the interphase cells showed Glγ‐tubulin localization at basal bodies, axonemes, and median body (Figure 2a). Staining with anti‐α‐tubulin antibodies indicated that 27% of the interphase cells did not have median bodies.

Interestingly, *Giardia* cells at dividing stages showed the localization of Glγ‐tubulin at basal bodies (Figure 2b). In cells at anaphase, Glγ‐tubulin was located at two spots, and the mitotic spindles stained with anti‐α‐tubulin antibodies were located between them. On the other hand, Glγ‐tubulin was detected outside of two nuclei of the telophase cells. During cytokinesis, it was found in basal bodies and axonemes of the two daughter cells. To confirm the localization of Glγ‐tubulin at MTOC (Lauwaet et al., 2011), anti‐Glcentrin antibodies were made, and their specificity was confirmed by an immunoreactive protein of 20 kDa in western blot analysis of *Giardia* extracts (data not shown). We then performed an IFA on dividing *Giardia* using antibodies for HA and Glcentrin (Figure 2c). These double‐stained *Giardia* cells with anti‐HA and anti‐Glcentrin antibodies showed co‐localization of Glγ‐tubulin and Glcentrin during cell division. These data suggested a possibility that Glγ‐tubulin localizes at MTOCs in dividing *Giardia*.

### 3.3 Effect of Glγ‐tubulin knockdown on cell division of *Giardia*

**Figure 3:**
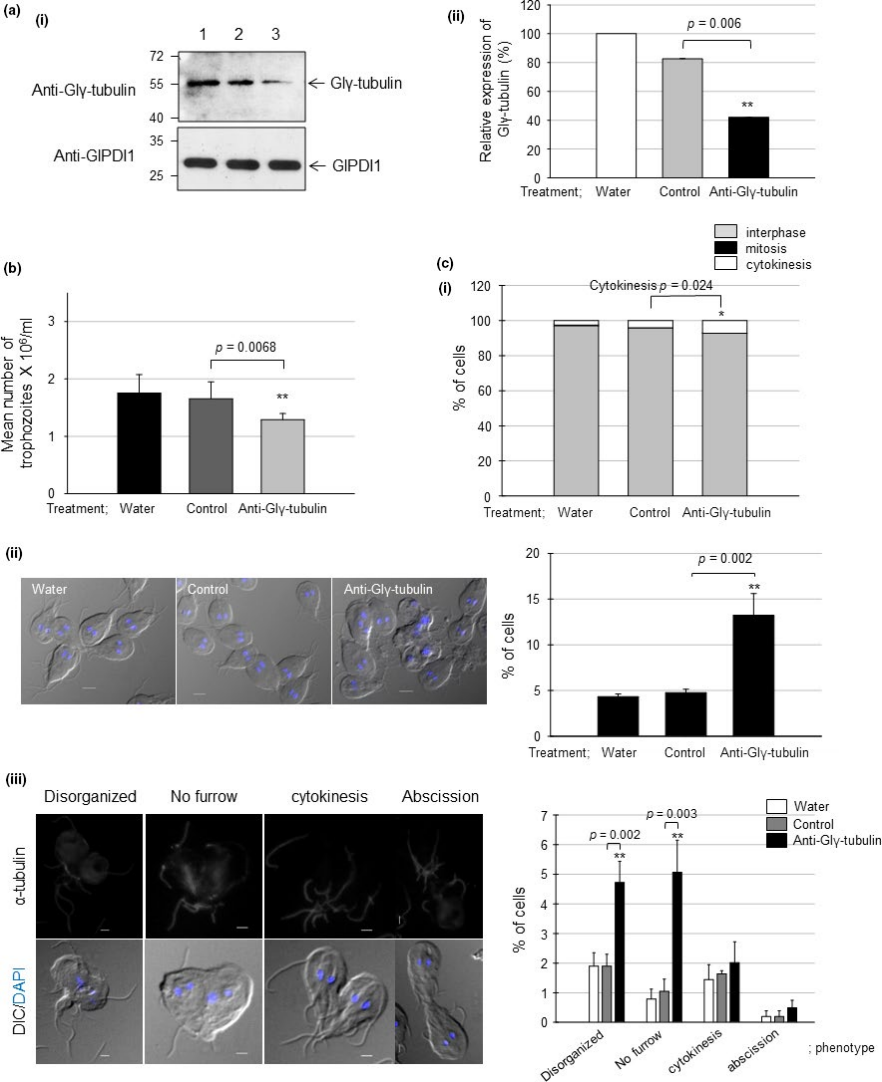
Effect of the morpholino‐mediated knockdown of Glγ‐tubulin on *Giardia* cell division. (a) *Giardia* trophozoites were collected at 24 hr after electroporation with water, a control morpholino, or an anti‐Glγ‐tubulin morpholino. (i) Extracts of these cells were reacted with anti‐Glγ‐tubulin or anti‐GlPDI antibodies. (ii) The abundance of each immunoreactive protein was quantified by densitometry and normalized to that of *Giardia* treated with water. Western blot analysis was performed on the extracts derived from at three independent knockdown experiments and quantified. One of western blots is presented as a representative. The quantified data are presented as the mean ± standard deviation from three independent experiments. (b) Effect of morpholino‐mediated knockdown of Glγ‐tubulin on *Giardia* growth. At 24 hr after the treatment with morpholino, the number of parasites per milliliter was determined using a hemocytometer. (c) Effect of anti‐Glγ‐tubulin morpholino on the cytokinesis of *Giardia* cells. (i) The cells were stained with 10% Giemsa solution and observed with a light microscope to count the numbers of cells at stage of interphase (gray columns), mitosis (closed columns), and cytokinesis (open columns). The cell number was determined by counting a least 500 cells per each condition. (ii) The cells were attached on coverslips and mounted in anti‐fade mounting medium with DAPI. To determine the numbers of cells with four or two nuclei, more than 300 cells per condition were counted. (iii) *Giardia* cells arrested at cytokinesis were categorized into the four phenotypes (disorganized pattern, no furrow, arrested cytokinesis, and failed abscission). Percentages of the cells with the four phenotypes are presented in the bar graph. Scale bar: 2 μm

To define the role of Glγ‐tubulin in *G. lamblia*, we designed an anti‐Glγ‐tubulin morpholino to block the translation of Glγ‐tubulin mRNAs (Table 1). A control morpholino (non‐specific oligomers) was also made and transfected into cells by electroporation (Table 1). Another set of *G. lamblia* was treated with sterile water instead of morpholino. These extracts at 24 hr post‐transfection were monitored to determine their intracellular levels of Glγ‐tubulin by western blot using anti‐Glγ‐tubulin antibodies (Figure 3a). Both cells treated with control or anti‐Glγ‐tubulin morpholino showed decreases in the amount of Glγ‐tubulin at 24 hr post‐transfection to 83% and 42%, respectively.

The growth of *Giardia* cells treated with water, the control morpholino, or the anti‐Glγ‐tubulin morpholino was determined (Figure 3b). After a 24 hr treatment with the anti‐Glγ‐tubulin morpholino, *Giardia* cells showed 24% growth inhibition as compared with the control morpholino‐treated cells (*p*‐value = 0.0068). On the contrary, the growth of *Giardia* cells treated with water was similar to that of the control morpholino‐treated cells.

The effect of Glγ‐tubulin knockdown on cell division was also determined by Giemsa staining of these cells to distinguish *Giardia* at different stages (i.e., interphase, mitosis, and cytokinesis) (Figure 3c(i)). A small proportion of the cells were at mitosis (0.4%–1%) or cytokinesis (2%–4%), whereas most of the cells were as interphase (96%–97%). In the case of *Giardia* cells treated with anti‐Glγ‐tubulin morpholino, the proportion of cytokinetic cells was increased to 7%, whereas that of *Giardia* cells treated with water or the control morpholino was 2% or 4%, respectively. An additional assay to determine the mitotic index also showed that proportion of cells with four nuclei increased from 4% to 5% for the control cells to 13% when the cells were treated with anti‐Glγ‐tubulin morpholino (Figure 3c(ii); *p*‐value = 0.002). These results indicated that the decreased expression of Glγ‐tubulin caused an arrest of cytokinesis, eventually leading to growth inhibition.

Since the treatment of anti‐Glγ‐tubulin morpholino increased number of *Giardia* cells with four nuclei, we examined the effect of Glγ‐tubulin knockdown on cytokinesis in detail as described (Hardin et al., 2017). They sub‐divided cytokinesis‐defective *Giardia* cells into four sequential phenotypes: the disorganized cells impertinent for cytokinesis, the cells defective in furrow formation, the arrested cells at cytokinesis, and the cells stopped at the abscission step. Compared to low level (2%) in control *Giardia* cells, the percentage of cells showing the disorganization was increased to 5% (Figure 3c(iii); *p*‐value = 0.002). In the same manner, dividing cells without furrow was increased from 1% (control cells) to 5% when the cells were treated with anti‐Glγ‐tubulin morpholino (*p*‐value = 0.003). On the contrary, the percentages of *Giardia* cells defective in the subsequent two steps, cytokinesis and abscission, were not significantly affected by Glγ‐tubulin knockdown.

### 3.4 Effect of Glγ‐tubulin knockdown on the cytoskeletal structure of *Giardia*

**Figure 4:**
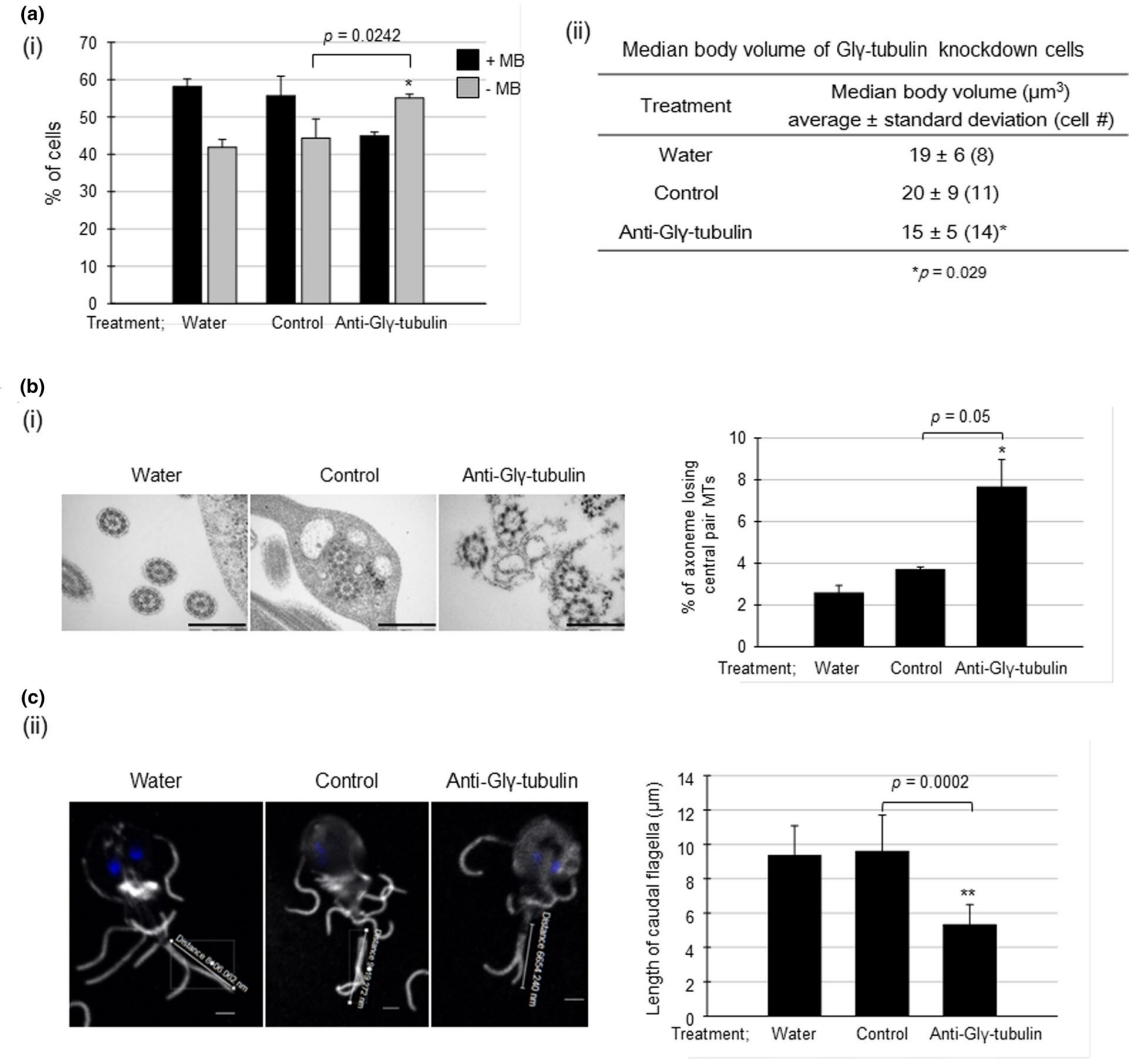
Effects of morpholino‐mediated knockdown of Glγ‐tubulin on the formation of median bodies and MT axonemes of *Giardia*. (a) Median body formation. (i) For *Giardia* cells treated with each condition, that is, water, a control morpholino, or an anti‐Glγ‐tubulin morpholino, the proportions of cells with a median body were determined by Giemsa staining. +MB: cells with the median body; ‐MB: cells without the median body. (ii) To measure the volume of the median bodies, the cells were stained with anti‐α‐tubulin antibodies (1:600), followed by a reaction with AlexaFlour 488‐conjugated anti‐mouse IgG (1:200). The stained cells were observed with a Zeiss LSM710 laser scanning confocal microscope. For the measurement of median body volume, images were analyzed using the Imaris (Bitplane) software. The significance of differences between each condition was evaluated by Student's *t* tests. Differences with *p*‐values of less than 0.05 were considered significant. (b) Flagella formation. (i) MT axoneme formation. *Giardia* cells treated with water, control morpholino, or anti‐Glγ‐tubulin morpholino were treated for TEM. In thin section of *G. lamblia* cells, the axonemes were scored as a canonical 9 + 2 MT axoneme or an axoneme losing the central pair of MTs. Scale bar: 5 μm. (ii) *G. lamblia* cells stained with anti‐α‐tubulin antibodies were also used to observe the effect of the anti‐Glγ‐tubulin morpholino on flagella formation. The length of external portion of the caudal flagella was measured by Zen 2012 software. Scale bar: 2 μm

*Giardia* cells treated with water, the control morpholino, or the anti‐Glγ‐tubulin morpholino and stained with Giemsa were also monitored to determine the presence of median body, which is a characteristic cytoskeletal structure of *G. lamblia* (Figure 4a(i)). In the control cells treated with water or the control morpholino, 42%–44% of cells were present without median body. Interestingly, the percentage of cells without a median body increased to 55% in the *G. lamblia* cells treated with anti‐Glγ‐tubulin morpholino.

The formation of the median body was also monitored by measuring their volume in the *Giardia* cells stained with anti‐α‐tubulin antibodies (Figure 4a(ii)). The volume of the median bodies of *Giardia* treated with anti‐Glγ‐tubulin morpholino significantly decreased to 75% of that of the control cells. On the contrary, the size of the median bodies in *Giardia* treated with water did not significantly differ from that in the *Giardia* cells treated with the control morpholino.

In addition to the median body, the formation of flagella was examined in *Giardia* treated with anti‐Glγ‐tubulin morpholino, especially the structure of the axoneme of the flagella (Figure 4b(i)). In the control cells, most of the flagella axonemes had a typical 9 + 2 structure, and only 3%–4% of the axonemes were devoid of the central pair MTs. In *Giardia* treated with anti‐Glγ‐tubulin morpholino, the proportion of abnormal axonemes lacking the central pair MTs increased to 8% (±1, *p*‐value = 0.05).

The subsequent experiment was performed to examine whether Glγ‐tubulin knockdown affected the length of the flagella in *G. lamblia*, especially the caudal flagella due to its higher technical accessibility than the other flagella (Figure 4b(ii)). *Giardia* cells stained with anti‐α‐tubulin antibodies were monitored to determine the length of the caudal flagella. We measured the length of caudal flagella from the external portion, because not easy to start from the axoneme portion inside the cells. The size of the caudal flagella decreased to 5 ± 1 µm in *Giardia* treated with the anti‐Glγ‐tubulin morpholino as compared with that of the control cells (9 ± 2 µm; *p*‐value = 0.0002).

### 3.5 Formation of γ‐TuSC in *G. lamblia*

γ‐Tubulin functions as a member of the γ‐TuSC along with γ‐tubulin complex proteins (GCP2 and GCP3). In higher eukaryotes, this γ‐TuSC forms more complicated complex called γ‐TuRC along with additional proteins, and this larger complex shows a more efficient MT nucleation activity (Lin, Neuner, & Schiebel, 2015). A database search for GCPs indicated the two open reading frames GL50803_17429 and GL50803_12057 for GlGCP2 and GlGCP3, respectively. However, no homologous proteins involved in γ‐TuRC formation were detected in the *G. lamblia* database.

**Figure 5:**
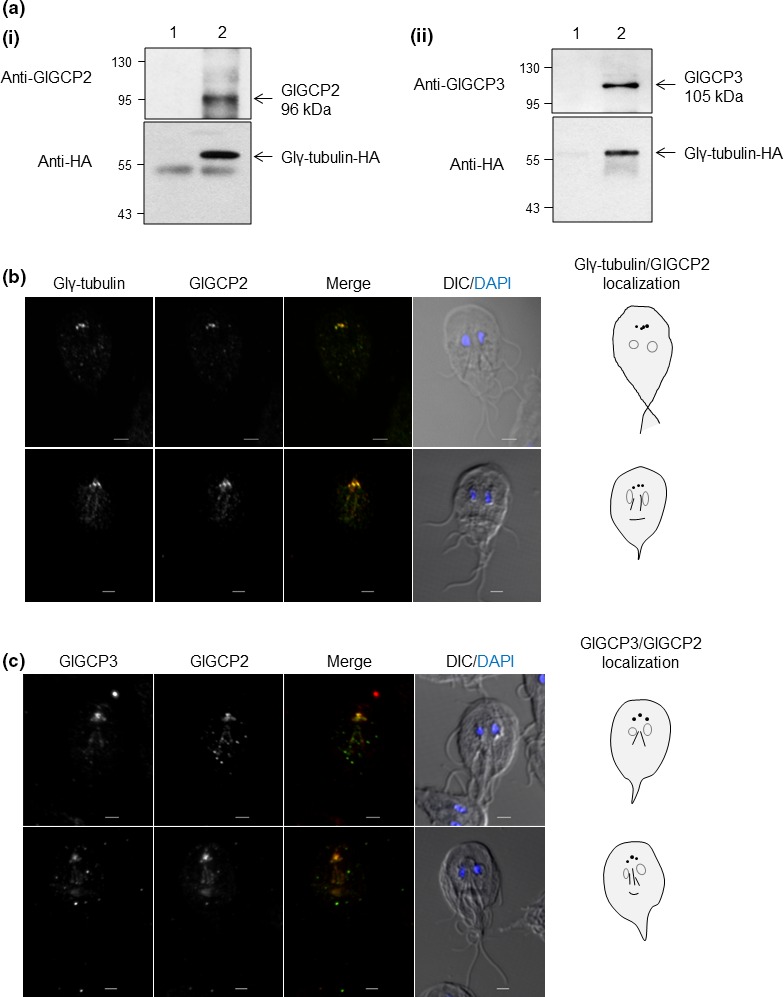
Interaction and co‐localization of the Glγ‐TuSC components in *Giardia*. (a) Co‐immunoprecipitation of GlGCP2 (i) and GlGCP3 (ii) with Glγ‐tubulin in *G. lamblia* expressing HA‐tagged Glγ‐tubulin. Extracts of *Giardia* expressing HA‐tagged Glγ‐tubulin were incubated with Sepharose bead‐conjugated anti‐mouse IgG (lane 1) or anti‐HA agarose beads at 4°C overnight (lane 2). Twenty micrograms of the eluted fraction was analyzed by western blot using anti‐GlGCPs or anti‐HA antibodies. (b) Co‐localization of GlGCP2 with Glγ‐tubulin. *Giardia* expressing HA‐tagged Glγ‐tubulin reacted with rat anti‐HA (1:100) and anti‐GlGCP2 antibodies (1:50). (c) Co‐localization of GlGCP3 with GlGCP2. *Giardia* carrying pGlGCP3HAX3.neo was stained with rat anti‐HA (1:50) and anti‐GlGCP2 antibodies (1:50). The cells were observed using an LSM710 laser scanning confocal microscope (Carl Zeiss). A DIC image was acquired to show the cell morphology. All the images are maximum‐intensity Z‐projections. Scale bar: 2 μm

In the following experiments, we examined whether these putative GlGCPs could form γ‐TuSC with Glγ‐tubulin (Figure 5a). Peptides with the sequences of GlGCP2 and GlGCP3 were made and used to generate specific antibodies for use in western blot analyses of *Giardia* extracts (data not shown). Cell extracts prepared from *Giardia* cells carrying pGlγ‐tubulinHAX3.pac were incubated with a resin coupled with anti‐HA antibodies. The resulting immunoprecipitates were analyzed for the presence of HA‐tagged Glγ‐tubulin, GlGCP2, and GlGCP3 using the specific antibodies (Figure 5a(i, ii), respectively). Both GlGCP2 and GlGCP3 were present along with HA‐tagged Glγ‐tubulin in the immunoprecipitates as a size of 96 and 105 kDa, respectively. When the same extracts were incubated with a resin‐coupled with anti‐mouse IgG, none of the GlGCP2, GlGCP3, and HA‐tagged Glγ‐tubulin was detected in the immunoprecipitation fraction.

To examine the intracellular localization of GlGCP2, *Giardia* cells carrying pGlγ‐tubulinHAX3.pac were stained with anti‐HA and anti‐GlGCP2 antibodies (Figure 5b). Both Glγ‐tubulin and GlGCP2 protein were present in the basal bodies, axonemes, and the median bodies of the interphase *Giardia* cells.

Since IFAs using anti‐GlGCP3 antibodies did not show any meaningful fluorescent signal under various reaction conditions, we constructed pGlGCP3HAX3part.neo (Supporting information Figure S1a) and then used to construct transgenic trophozoites expressing the HA‐tagged GlGCP3′. The expression of the HA‐tagged GlGCP3′ was shown as an immunoreactive protein band of ~81 kDa in a western blot analysis of the resulting *G. lamblia* extracts (Supporting information Figure S1b). The intracellular level of GlPDI1 was monitored as a loading control.

Additional IFAs were performed to examine the localization of both GCP2 and GCP3 in the *Giardia* cells at the interphase (Figure 5c). *Giardia* trophozoites expressing the HA‐tagged GlGCP3 were stained with anti‐HA and anti‐GlGCP2 antibodies. Both GlGCP2 and GlGCP3 were found in the basal bodies, axonemes, and the median bodies of the *Giardia* cells.

### 3.6 Phenotypes of GlGCP2 and GlGCP3 knockdown in *G. lamblia*

**Figure 6:**
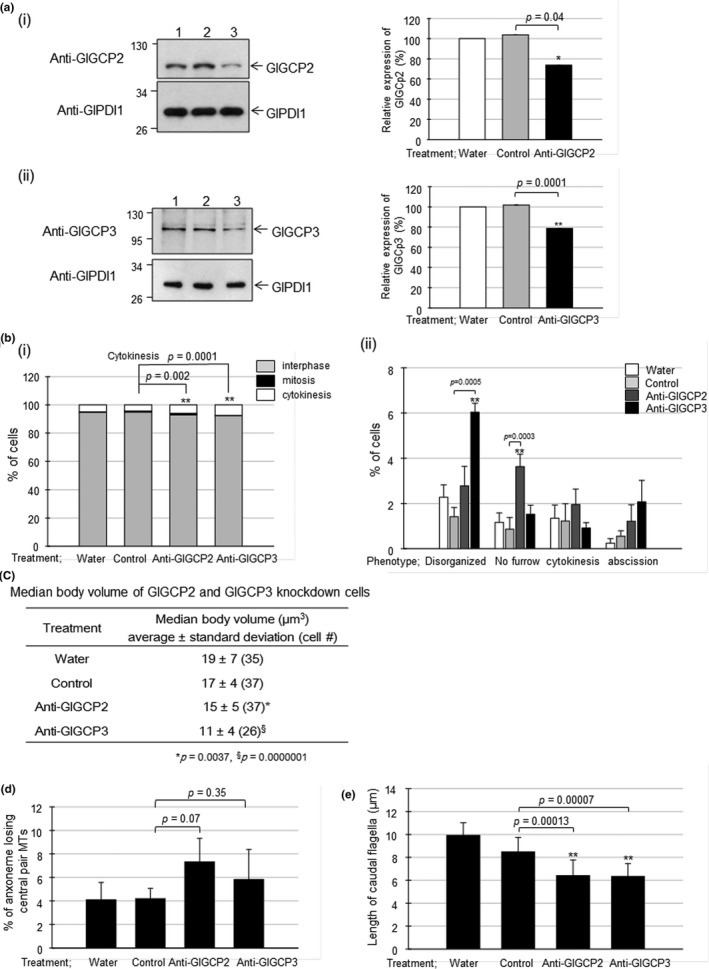
Phenotypes of morpholino‐mediated knockdown of GlGCP2 and GlGCP3 in *Giardia*. *Giardia* trophozoites were collected at 24 hr after electroporation with water, a control morpholino, or anti‐GlGCPs morpholino. (a) Decreased expression of GlGCP2 (i) and GlGCP3 (ii) in *Giardia* cells treated with anti‐GlGCPs‐morpholino. Western blot analysis was performed on the extracts derived from at three independent knockdown experiments and quantified. One of western blots is presented as a representative. The quantified data are presented as the mean ± standard deviation from three independent experiments. (b) Effects of GlGCPs knockdown on cytokinesis in anti‐GlGCPs morpholino‐treated *Giardia* cells. (i) The cells were stained with 10% Giemsa solution and then observed to count the numbers of cells in the stage of interphase (gray columns), mitosis (closed columns), and cytokinesis (open columns). The numbers of cells were counted in over 500 cells per each condition. (ii) The cells with four nuclei were classified into four groups according to their morphology: the disorganized cells, the cells without furrows, the cells at cytokinesis, and the cell arrested at the abscission. (c) Effects of GlGCP knockdown on median body volume. Each cell was stained with anti‐α‐tubulin antibodies. The stained cells were observed with a Zeiss LSM710 laser scanning confocal microscope. For the measurement of median body volume, images were analyzed by using the Imaris (Bitplane) software. (d) The proportions of cells with an intact axoneme or an axoneme losing the central pair of MTs. MT axonemes from cells treated with water, a control, or anti‐GlGCP morpholino were determined using transmission electron microscopy. (e) *G. lamblia* cells stained with anti‐α‐tubulin antibodies were also used to observe the effects of the anti‐GlGCP morpholino on length of the caudal flagella by using Zen 2012 software

If Glγ‐tubulin functions by forming Glγ‐TuSC with GlGCP2 and GlGCP3, the knockdown of GlGCP2 and GlGCP3 using anti‐GlGCP2 and anti‐GlGCP3 morpholino would also be expected to result in a phenotype similar to that of *Giardia* cells with a decreased expression of Glγ‐tubulin. Most of all, extracts of at 24 hr post‐transfection were prepared to monitor for their intracellular levels of GlGCP2 or GlGCP3 by western blots using its specific antibodies (Figure 6a(i,ii), respectively). The cells treated with anti‐GlGCP2 and anti‐GlGCP3 morpholino showed decreases in the amounts of GlGCP2 or GlGCP3 at 24 hr post‐transfection to 74% and 79% of the amounts in the control group, respectively.

The effect of cell division in anti‐GlGCPs morpholino‐treated cells was determined by Giemsa staining to distinguish *Giardia* at different stages (Figure 6b(i)). In the case of *Giardia* cells treated with anti‐GlGCP2 or anti‐GlGCP3 morpholino, the proportion of arrested cells at cytokinesis was increased to 6% or 7%, respectively, whereas that of *Giardia* cells treated with the control morpholino was 4% (*p*‐value = 0.002 and 0.0001 for GlGCP2 and GlGCP3 knockdown, respectively). Effect of GlGCPs knockdown on *Giardia* cytokinesis was further analyzed with respect to the four sequential phenotypes indicating cytokinesis defects (Figure 6b(ii)). In case of the GlGCP2 knockdown cells, the percentage of cells without furrows was significantly increased to 4% compared with those of the control cells (1%, *p*‐value = 0.0003). More disorganized cells for cytokinesis (6%) were detected in the GlGCP3 knockdown group than the control groups (1%–2%, *p*‐value = 0.0005). Like the Glγ‐tubulin knockdown cells, they did not show any increase in the percentages of cells arrested at cytokinesis and abscission.

*Giardia* cells stained with anti‐α‐tubulin were monitored to determine the volume of their median bodies (Figure 6c). The volumes of the median bodies in the control groups treated with water or the control morpholino were not significantly different. The volumes of the median bodies of *Giardia* treated with the anti‐GlGCP2 or anti‐GlGCP3 morpholino decreased to 85% or 66% of that of the control cells, respectively.

The flagella were examined in *Giardia* treated with anti‐GlGCP morpholino, especially the structure of the axoneme of the flagella (Figure 6d). Abnormal axonemes without the central pair MTs were found at 6%–7% of *Giardia* treated with anti‐GlGCP2 or anti‐GlGCP3 morpholino. The proportion of cells with an abnormal axoneme was 4% in the control cells. However, these differences among the groups were not statistically significant (*p*‐value = 0.07–0.35).

Thus, we improved this assay by differentiating the affected one among the four pairs of *Giardia* flagella. The putative locations of the axonemes for the anterior, caudal, posterolateral, and ventral flagella were indicated in the cartoon (Supporting information Figure S2a), and they were used to monitor the TEM images of various *Giardia* cells (Figure 3b–d). In control cells, the prevalence rates of the affected axonemes were 2%–5% and 0%–3% for the posterolateral and the ventral flagella. We could not observe any affected axoneme which was definitely identified as the anterior or the caudal flagella. Treatment of *Giardia* cells with anti‐Glγ‐tubulin morpholino increased the percentages of the affected posterolateral and ventral axonemes to 13 and 11, respectively (Supporting information Figure S2b). Still, none of the anterior or the caudal axonemes was found to be devoid of the central pair MTs. In case of *Giardia* cells treated with anti‐GlGCP2, a little increase to 5% was detected only in the axonemes of posterolateral flagella (Supporting information Figure S2c). On the other hand, the axonemes of both posterolateral and ventral flagella were more affected by the treatment of anti‐GlGCP3 morpholino (13% and 5%, respectively).

In addition, the length of the caudal flagella was monitored in *G. lamblia* treated with anti‐GlGCP morpholino (Figure 6e). The lengths of the caudal flagella of the control cells were 10 ± 1 and 9 ± 1 µm, and this measurement decreased to 6 ± 1 µm upon the knockdown of GlGCP2 and GlGCP3 (*p*‐values = 0.00013 and 0.00007).

### 3.7 Relationship of Glγ‐TuSC with GlEB1 in the median body and flagella biogenesis

**Figure 7:**
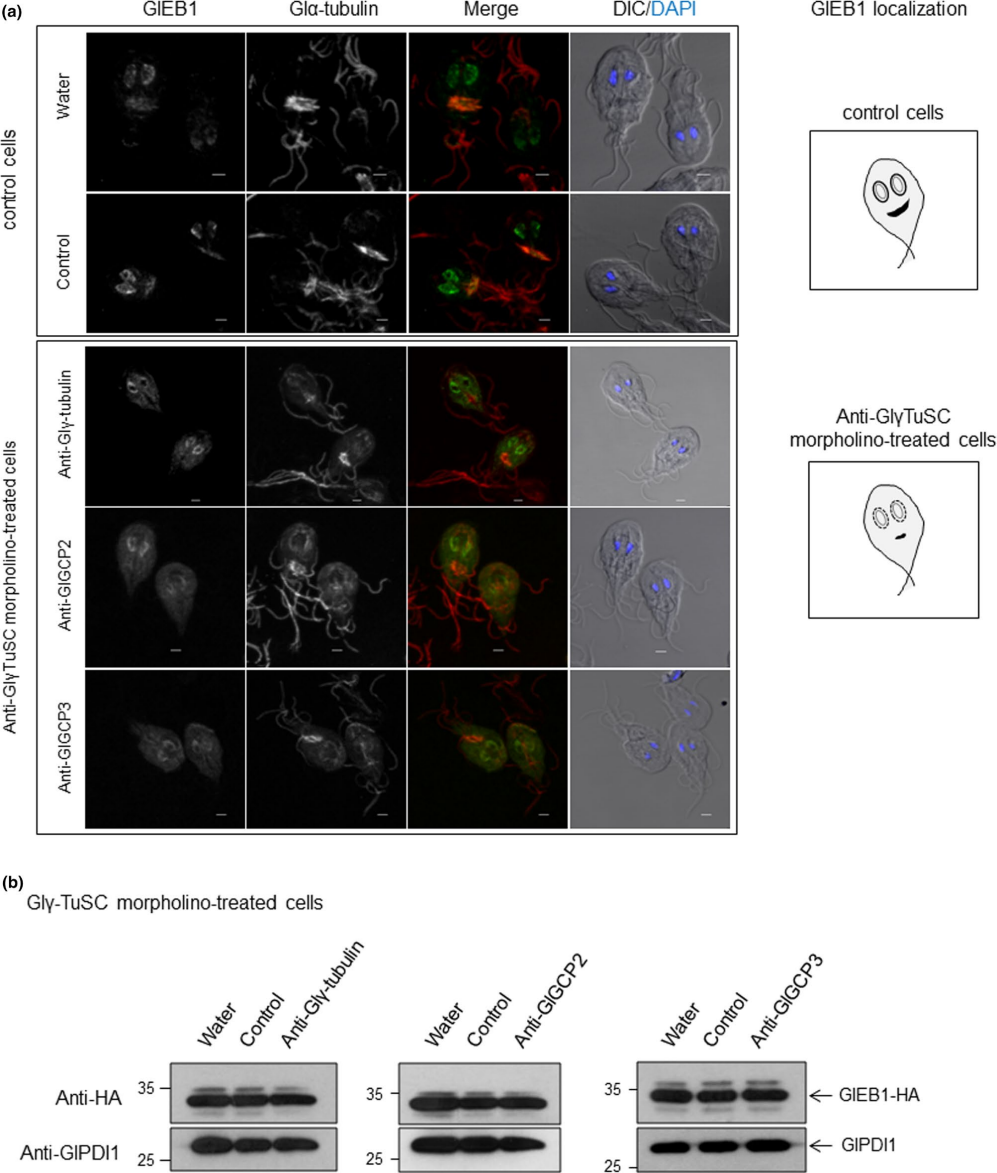
Localization of GlEB1 in Glγ‐TuSC‐knockdown cells. (a) Localization of GlEB1 in *Giardia* cells treated with water, a control, or an anti‐Glγ‐TuSC morpholino. HA‐tagged GlEB1 expressing cells were collected at 24 hr after electroporation. The cells were then reacted with rat anti‐HA (1:50) and anti‐α‐tubulin antibodies. They were incubated with AlexaFluor 488‐conjugated anti‐rat IgG (1:100) and AlexaFluor 564‐conjugated anti‐mouse IgG (1:100). All the images are maximum‐intensity Z‐projections. Scale bar: 2 μm. (b) The intracellular level of GlEB1 were analyzed by western blot analysis of Glγ‐TuSC knockdown cells with anti‐HA antibodies. HA‐tagged GlEB1 expressing cells were collected at 24 hr after electroporation with water, a control, or an anti‐Glγ‐TuSC morpholino. The same extracts were analyzed for GlPDI1 as a loading control

In the subsequent experiment, we examined whether Glγ‐tubulin affects the function of GlEB1. To do this, the *Giardia* cells expressing HA‐tagged GlEB1 were treated with anti‐Glγ‐TuSC morpholino, that is, anti‐Glγtubulin, anti‐GlCGP2, or anti‐GlGCP3 morpholino, and then double‐stained with anti‐HA and anti‐α‐tubulin antibodies to determine the localization of GlEB1 and MTs (Figure 7a). In control cells, we could observe the localization of GlEB1 at the nuclear membranes and the median bodies. The localization of GlEB1 in the nuclear membrane as well as in the median body was not distinct in Glγ‐TuSC knockdown cells. A western blot analysis in these cells showed that the expression level of GlEB1 was not changed in the Glγ‐TuSC knockdown cells (Figure 7b). This result suggests the possibility that Glγ‐TuSC may be required for the correct positioning of GlEB1 in *Giardia*.

**Figure 8:**
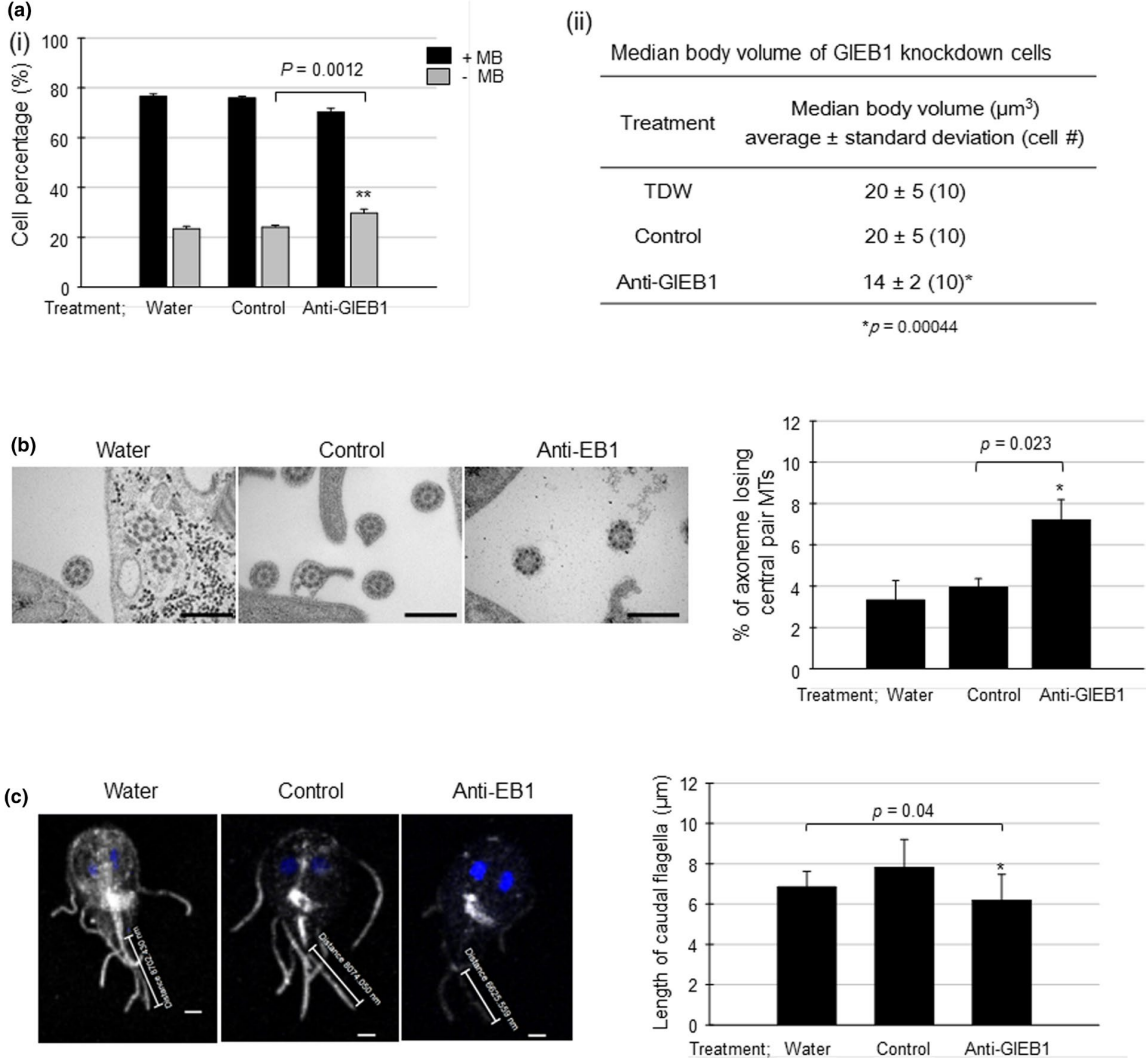
Effect of morpholino‐mediated knockdown of GlEB1 on the formation of the median body, and MT axoneme, and caudal flagella of *Giardia*. (a) Median body formation. (i) For *Giardia* cells treated with water, control morpholino, or anti‐GlEB1 morpholino, the proportion of cells with a median body was determined by Giemsa staining. +MB: cells with the median body; ‐MB: cells without the median body. (ii) To measure the volume of median body, the cells were stained with anti‐α‐tubulin antibodies (1:600), followed by a reaction with AlexaFlour 488‐conjugated anti‐mouse IgG (1:200). The stained cells were observed with a Zeiss LSM710 laser scanning confocal microscope. For the measurement of median body volume, images were measured by using the Imaris (Bitplane) software. The significance of differences between the experimental conditions was evaluated by Student's *t* tests. Differences with *p*‐values of <0.05 were considered significant. (b) MT axoneme formation. MT axonemes from cells treated with water, a control, or an anti‐GlEB1 morpholino were analyzed by transmission electron microscopy. The effect of GlEB1 knockdown on MT axoneme formation was presented as the percentage of cells with an axoneme losing the central pair of MTs. Scale bar: 5 μm. (c) *G. lamblia* cells stained with anti‐α‐tubulin antibodies were also used to observe the effect of anti‐GlEB1 morpholino on the length of the caudal flagella by using Zen 2012 software. Scale bar: 2 μm

Therefore, we examined the phenotype of the GlEB1 knockdown cells with respect to the formation of the flagella and median body in the following experiments. A previous study indicated that knockdown of the GlEB1 resulted in a cytokinesis defect (Kim et al., 2017, 2014 ). GlEB1 knockdown also resulted in an increased proportion of *Giardia* cells without a median body (from 24% to 30% *p*‐value = 0.0012; Figure 8a(i)). In addition, the volume of the median bodies was affected in GlEB1‐knockdown cells (Figure 8a(ii)). A TEM analysis of *Giardia* cells with reduced expression level of GlEB1 also showed a higher frequency of axonemes missing the central pair MTs (7%) than in the cells treated with control morpholino (4%) (*p*‐value = 0.023) (Figure 8b). A detailed analysis on types of affected flagella revealed that posterolateral and ventral flagella lost the central MT pair, but the anterior and caudal flagella did not show any defect (Supporting information Figure S2d). With respect to the caudal flagella, the length of these flagella was slightly affected by GlEB1 knockdown in *G. lamblia* and decreased from 7 ± 1 µm in the control group to 6 ± 1 µm in the GlEB1‐knockdown group (*p*‐value = 0.04) (Figure 8c).

## 4 DISCUSSION

In *S. cerevisiae*, a mutant of γ‐tubulin was defective in the proper recruitment of the Kar9p‐Bim1p complex at the MT tips and only the overexpression of Bim1p restored this mutant phenotype (Cuschieri et al., 2006). Although a direct association between these two proteins has not been shown, their study suggested a functional relationship between Bim1p and γ‐tubulin. In the current study, we demonstrated a direct interaction between *G. lamblia* γ‐tubulin and GlEB1, which is the Bim1p ortholog of *G. lamblia*, through in vivo and in vitro assays (Figure 1). This interaction seems to occur in *G. lamblia* as shown by the co‐immunoprecipitation of GlEB1 with HA‐tagged Glγ‐tubulin (Figure 1d(iii)).

γ‐Tubulin plays roles in the nucleation and regulation of MT assembly; therefore, it localizes at MTOCs such as centrosomes or spindle pole bodies (Joshi, Palacios, McNamara, & Cleveland, 1992; Wiese & Zheng, 2006). In *Trypanosoma brucei*, γ‐tubulin localizes at basal bodies, which function as an MTOC for the nucleation of flagella/cilia and functions as spindle poles during cell division (Scott, Sherwin, & Gull, 1997; Zhou & Li, 2015). In *Giardia*, the localization of γ‐tubulin at the basal bodies was reported in a study using monoclonal human γ‐tubulin antibodies (Nohynková et al., 2000) and a proteomic analysis of the basal bodies (Davids, Shah, Yates, & Gillin, 2011). In this study, we constructed transgenic *Giardia* expressing HA‐tagged Glγ‐tubulin and observed the localization of Glγ‐tubulin at the basal bodies, axonemes, and median bodies in the interphase trophozoites (Figure 2a). Interestingly, one‐fourth of the interphase cells were found to be devoid of the median body, in which Glγ‐tubulin was present at the basal bodies. In the rest of the interphase cells, they had a median body at which Glγ‐tubulin was co‐localized with MTs (Figure 2a). This observation is consistent with the study performed by Horlock‐Roberts et al. (2017), in which they found that the size of median bodies varies according to the stages of *Giardia* cell cycle. That is, *Giardia* cells at G1 phase are present without median bodies, whereas they have bigger median bodies at G2 phase. Thus, our results showing the localization of Glγ‐tubulin at the median bodies and its plausible role in the formation of median body via the knockdown of Glγ‐tubulin cells should be interpreted with a caution (Figure 4a).

For MT nucleation, γ‐tubulin must form a complex with GCPs (Gull, 1999). The most basic MT nucleation machinery is the γ‐TuSC complex, which is composed of γ‐tubulin and two GCPs (Spc97p and Spc98 in budding yeast; GCP2 and GCP3 in humans; Lin et al., 2015). A database search and proteomic analysis of basal bodies (Davids et al., 2011) indicated that *Giardia* only has γ‐TuSC components, that is, Glγ‐tubulin, GlGCP2, and GlGCP3. The presence of γ‐TuSC in *G. lamblia* was shown in co‐immunoprecipitation experiments in which Glγ‐tubulin was co‐sedimented with putative GlGCP2 and GlGCP3 (Figure 5a(i,ii), respectively). In addition, the knockdown of GlGCP2 or GlGCP3 resulted in identical phenotype to the knockdown of Glγ‐tubulin with respect to reduced volume of the median bodies (Figure 6c). The median bodies are known as a unique structure in *Giardia* that may function as a MTOC and a reservoir of polymerized MTs (Piva & Benchimol, 2004). In *Giardia*, the ectopic expression of mutant kinesin‐13, a motor protein depolymerizing MTs at the plus and minus ends, caused significant decreases in the median body volume and resulted in mitotic defects (Dawson et al., 2007).

Our study also suggested that *Giardia* has a canonical γ‐TuSC, and Glγ‐TuSC is important in median body formation in *Giardia*. Our understanding of the role and assembly of this unique structure should be improved in order to reveal the cell cycle machinery operating in *G. lamblia*. In addition, Glγ‐TuSC seems to be important for cytokinesis in *G. lamblia* as demonstrated in the knockdown experiments of Glγ‐TuSC components, Glγ‐tubulin (Figure 3 for Glγ‐tubulin), GlGCP2, and GlGCP3 (Figure 6 for GlGCP2 and GlGCP3). Upon close observation on *Giardia* treated with anti‐Glγ‐TuSC morpholino, the disorganized cells and the cells without a furrow were more frequently found than the other two phenotypes, the cells undergoing cytokinesis and the cells arrested at abscission. This result implied that Glγ‐TuSC play a role in the early step of cytokinesis.

Interestingly, *Giardia* cells with a decreased level of Glγ‐tubulin frequently lost the central pair MTs in the flagella axonemes (Figure 4b(i)) and had shorter caudal flagella than the control cells (Figure 4b(ii)). The knockdown of GlGCP2 or GlGCP3 also increased the incidence of aberrant MT axonemes from the canonical 9 + 2 MT axonemes; however, the differences were not statistically significant (Figure 6d). A decreased expression of both GlGCP2 and GlGCP3 resulted in shorter caudal flagella than the control cells (Figure 6e). The less dramatic effects of GCP2 and GCP3 knockdown on MT axonemes than that of Glγ‐tubulin may stem from a lesser degree of inhibition by the anti‐GlGCP2 and anti‐GlGCP3 morpholino (Figure 6a(i,ii), respectively) as compared with the anti‐Glγ‐tubulin morpholino (Figure 3a). Thus, this investigation showed for the first time that the knockdown of Glγ‐TuSC in *G. lamblia* resulted in aberrant axonemes with the normal nine outer doublet MTs without the central pair MTs. Decreasing the expression of *T. brucei* γ‐tubulin or *T. brucei* GCPs (TbGCP2 and TbGCP3) by RNA interference also resulted in axonemes losing the central pair MTs and defects in the biogenesis of new flagellum (McKean, Baines, Vaughan, & Gull, 2003; Zhou & Li, 2015). In *G. lamblia*, flagella had been shown to play a role in cytokinesis through knockdown of paralyzed flagella 16 protein, which is associated with the central pair MTs of the flagella (Hardin et al., 2017).

Based on the previous investigation (Nohynková et al., 2000), a cartoon was made to predict the position of the axonemes of eight flagella (Supporting information Figure S2a) and used to differentiate the affected flagella axonemes in our TEM figures (Supporting information Figure S2b,c). The axonemes losing the central pair MTs were mainly identified as the ventral and the posterolateral flagella. This result supports a model that the ventral and posterolateral flagella are newly made in the daughter cells, whereas the caudal and anterior flagella are derived from the parental *Giardia* cells (Nohynková, Tumová, & Kulda, 2006). However, Glγ‐TuSC also plays a role in biogenesis of the caudal and anterior flagella, even though the effect of knockdown of Glγ‐TuSC is much less than that on the other two flagella. It is supported by the reduced length of caudal flagella in the Glγ‐TuSC knockdown cells (Figure 4b(ii,e)) as well as in the GlEB1‐knockdown cells (Figure 8c). Thus, it is possible that the cytokinesis defects in the Glγ‐TuSC‐depleted or GlEB1‐depleted cells are caused from malfunction of the four pairs of flagella. This idea is in a good agreement with the study of Hardin et al. (2017). Through in vivo image analysis of *Giardia* cells expressing fluorescence‐labeled MTs, they assigned the role in cytokinesis to all of four pairs of flagella. That is, the caudal flagella provide the initial force to orient the daughter cells into an opposite direction, whereas beating of the anterior flagella give the propulsion of cytokinesis. The other two new synthesized flagella, the posterolateral and the ventral flagella, are proposed to be involved in the furrow formation.

One of the interesting findings in this study is the relationship of Glγ‐tubulin with GlEB1. An IFA of GlEB1 in Glγ‐TuSC knockdown cells demonstrated that the localization of GlEB1 was altered (Figure 7a), whereas there was no change in the expression level of GlEB1 (Figure 7b). This result suggests the possibility that Glγ‐TuSC is essential for GlEB1 to localize in its correct position in *G. lamblia*. We then examined whether the knockdown of GlEB1 produced a similar phenotype to that of Glγ‐TuSC knockdown. As expected, the decreased expression of GlEB1 resulted in an increased number of cells without the median body, a decreased median body volume, aberrant axonemes losing of the central pair MTs, and shortening of the caudal flagella (Figure 8). It has been reported in other organisms that mutations or deficiencies in γ‐tubulin or GCPs affect the dynamics of plus‐end microtubules (Bouissou et al., 2009; Paluh et al., 2000; Vogel et al., 2001; Zimmerman & Chang, 2005). The mutant form of γ‐tubulin alters the distribution of the plus‐end‐tracking protein Bim1p, which is homolog to EB1, in *S. cerevisiae* (Cuschieri et al., 2006) and the depletion of GCPs also affects EB1 in *Drosophila* (Bouissou et al., 2014). An explanation for γ‐tubulin interaction with plus‐end protein MT dynamic is that γ‐tubulin complexes at MTOCs bind catastrophe or rescue factors or the motor molecules that transport them while there is constant bidirectional transport along microtubules (Oakleya, Paolilloa, & Zheng, 2015). Further experiments should be performed to observe the changes in the distribution of Glγ‐TuSC and GlEB1 during the cell cycle of *Giardia*.

Our investigation demonstrated that *Giardia* has a canonical Glγ‐TuSC, which plays a role in MT nucleation for median body formation and flagella biogenesis, and that GlEB1 may be involved in this process.

## CONFLICT OF INTEREST

The authors declare that there is no conflict of interest.

## AUTHORS CONTRIBUTION

Juri Kim and Soon‐Jung Park conceived and designed the experiments. Juri Kim performed the experiments. Juri Kim and Soon‐Jung Park analyzed the data and wrote the manuscript.

## ETHICS STATEMENT

Not required

## Data Availability

All data are included within the manuscript.
