# Supplementary figures and images for: Roles of end‐binding 1 protein and gamma‐tubulin small complex in cytokinesis and flagella formation of Giardia lamblia
Source: Microbiologyopen. 2018 Oct 14;8(6):e00748. doi: 10.1002/mbo3.748 (PMC6562232; doi:10.1002/mbo3.748)

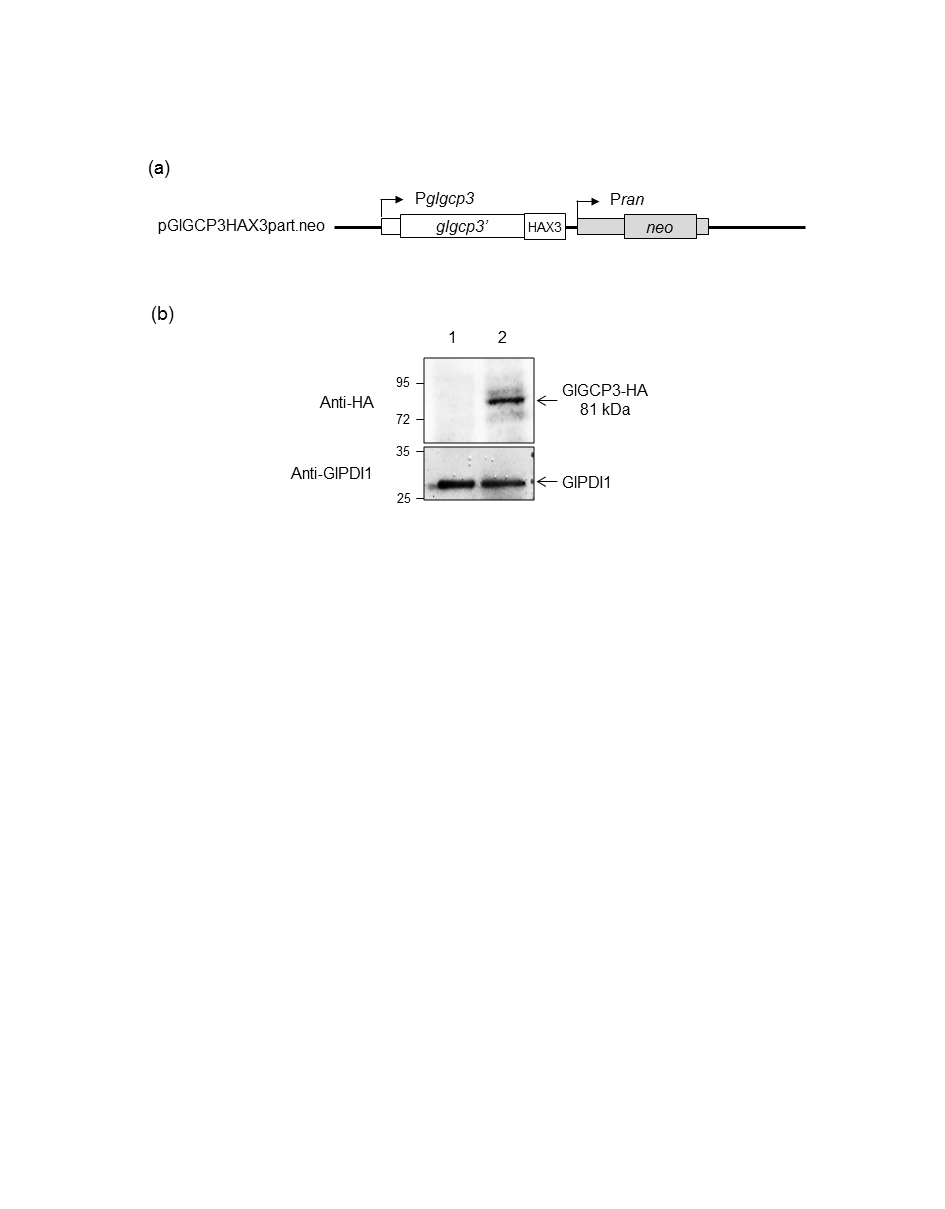

Supplement: Supplementary file 1 [file MBO3-8-e00748-s001.TIF]

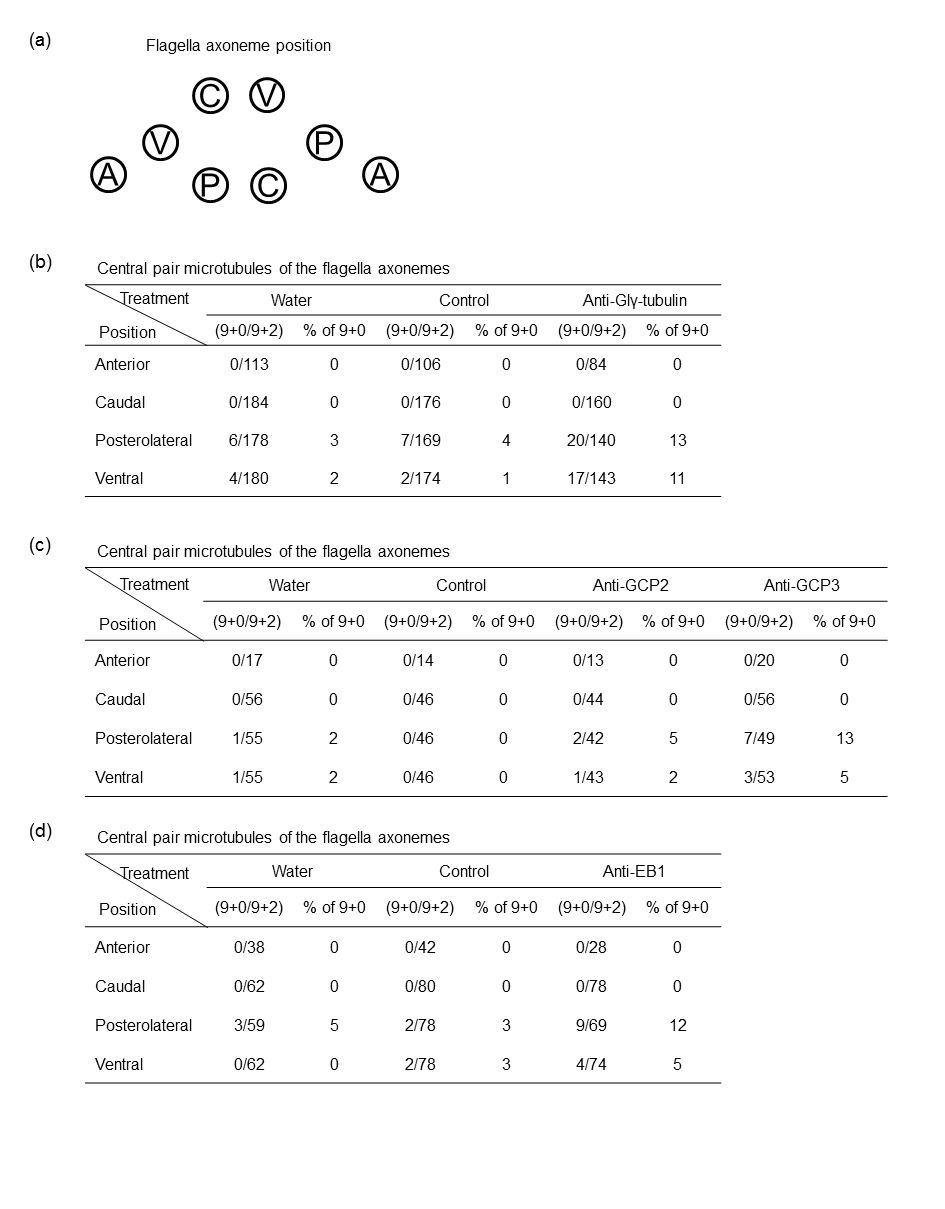

Supplement: Supplementary file 2 [file MBO3-8-e00748-s002.TIF]
