# Supplementary material for: Roles of end‐binding 1 protein and gamma‐tubulin small complex in cytokinesis and flagella formation of Giardia lamblia
Source: Microbiologyopen. 2018 Oct 14;8(6):e00748. doi: 10.1002/mbo3.748 (PMC6562232; doi:10.1002/mbo3.748)
Supplement: Supplementary file 3 [file MBO3-8-e00748-s003.docx]

**SUPPLEMENTARY FIGURE 1**

Construction of *G. lamblia* expressing HA-tagged partial GlGCP3. (a) A schematic diagram of the plasmid pGlGCP3HAX3part.neo. GlGCP3 is expressed from its own promoter, P*glgcp3*, as a HA-tagged form (HAX3). Transfected cells are selected by G418 resistance by the *neo* gene expressed by the P*ran* promoter, a strong promoter of the ras-related nuclear protein gene. (b) The expression of HA-tagged GlGCP3 was confirmed by western blot analysis. Extracts were prepared from *G. lamblia* containing pRAN.neo (lane 1), or pGlGCP3HAX3part.neo (lane 2). The membrane was reacted with monoclonal mouse anti-HA (1:1,000). After deprobing in the stripping buffer, the membrane was incubated with polyclonal rat antibodies specific to PDI1 of *G. lamblia* (1:10,000) as loading control.

**SUPPLEMENTARY FIGURE 2**

Effect of morpholino-mediated knockdown of Glγ-TuSC and GlEB1 on flagella formation in *Giardia*. TEM images of various *Giardia* cells (controls, the cells treated with anti-Glγ-tubulin, anti-GlGCP2, anti-GlGCP3, or anti-GlEB1 morpholino) were at first examined for the presence of axonemes losing the central pair MTs, and then they were specified on the type among the four pairs of *Giardia* flagella. (a) A cartoon showing the putative locations of the axonemes for the anterior, caudal, posterolateral, and ventral flagella in the transverse TEM section of *Giardia* trophozoites. (b) Effect of anti-Glγ-tubulin morpholino on central pair MTs of the flagella axonemes. (c) Effect of anti-GlGCPs morpholino on central pair MTs of the flagella axonemes. (d) Effect of anti-GlEB1 morpholino on central pair MTs of the flagella axonemes.
